# Supplementary material for: Minimal exposure of lipid II cycle intermediates triggers cell wall antibiotic resistance
Source: Nat Commun. 2019 Jun 21;10:2733. doi: 10.1038/s41467-019-10673-4 (PMC6588590; doi:10.1038/s41467-019-10673-4)
Supplement: Supplementary file 1 — Supplementary Information [file 41467_2019_10673_MOESM1_ESM.pdf]

Supplementary Information

Minimal exposure of lipid II cycle intermediates triggers cell wall antibiotic resistance

Piepenbreier *et al*.

Content

Supplementary Note 1

Supplementary Figure 1: Calibration of the theoretical model of the lipid II cycle in *E. coli*.

Supplementary Figure 2: Different multimeric binding scenarios lead to cooperative antibiotic-target-interaction.

Supplementary Figure 3: Theory predicts a moderate *in vivo* efficacy gap for friulimicin and a more pronounced difference between IC_{50} and K_D for vancomycin and ramoplanin.

Supplementary Figure 4: Model predictions of the *in vivo* efficacy gap are robust against variations in the model parameters.

Supplementary Figure 5. Sensitivity of predicted IC_{50} values on changes of experimentally determined model parameters.

Supplementary Table 1: Model calibration.

Supplementary Table 2: Parameter fitting and validation.

Supplementary Table 3: Comparison between *E. coli* and *B. subtilis*.

Supplementary Table 4: Antibiotic activity in *B. subtilis*.

Supplementary References

Supplementary Note 1

Quantitative considerations on peptidoglycan synthesis in *E. coli*

The rate of peptidoglycan (PG) production in the studied organisms was estimated based on experimentally determined total number of peptidoglycan monomers per cell. The cell wall of *E. coli* comprises of ~1.5 layers of PG (1), containing 3.5 x 10^6 PG monomers (GlcNAc-MurNAc-pentapeptides) in total (2). However, a significant portion δ of PG is degraded by hydrolases during cell growth. Accordingly, since a cell wall turnover of ~50% was observed (3, 4), within one doubling time a total number of 5.25 x 10^6 monomers of PG has to be translocated across the cytoplasmic membrane in order to satisfy the demand of PG of one *E. coli* cell. Hence, for a doubling time T_D of 36 min (corresponding to the measured amount of PG monomers (2)), the rate of PG synthesis is given by:

$$j_{\mathrm{PG}} = (1 + \delta) \times (\text{PG monomers per cell}) \times \frac{\ln(2)}{T_{\mathrm{D}}}$$

$$= 3.5 \times 10^6 \text{ PG monomers cell}^{-1} \times 1.5 \times \frac{\ln(2)}{36 \text{ min}} = 1.01 \times 10^5 \text{ PG monomers cell}^{-1}\text{min}^{-1}$$

Given that the lipid II cycle (Fig. 1a) is the major pathway of PG synthesis, the transition time of individual carriers through all states of the cycle determines the rate of PG monomer transport across the cytoplasmic membrane. We derived this transition time from assuming a closed-loop system of cyclic reactions – showing subsequently the validity of this assumption by assessing the relevance of *de novo* synthesis of individual carriers (Supplementary Fig. 1a). In such a closed-loop system, all substrate pools of the individual reactions equilibrate such that the individual fluxes - defined as the actual number of reactions occurring per time interval - are identical and, in particular, equal to the PG synthesis rate. Accordingly, by transitioning through the different states of the cycle, the dwell time of individual intermediates in every state differs, based on the speed of the subsequent enzymatic reaction (Supplementary Fig. 1b). More precisely, in highly efficient enzymatic reactions, catalyzed by enzymes with elevated catalytic rates or with high enzyme abundances, a substrate is converted into the product state much faster and, consequently, dwells much shorter in the substrate state, than in less efficient reactions. This governs the distribution of the pool levels within the cycle and leads to small substrate pools for fast enzymatic reactions and an accumulation of substrates for reactions catalyzed by slower or less enzymes (compare Supplementary Table 1a). Hence, the time one carrier molecule needs to complete a full round in the lipid II cycle was calculated from the dwell times within every intermediate state (Supplementary Fig. 1b). Considering the simplest scenario, the flux from one state into the next one, j_i, depends on the substrate levels (S_i) and the speed of the reactions (k_i) and is equal to the PG synthesis rate j_{PG}, as mentioned before

$$j_{\mathrm{i}} = j_{\mathrm{PG}} \sim k_{\mathrm{i}}[S_{\mathrm{i}}]$$

This assumption of a proportional dependency of the flux from the substrate pools is valid in the case of first order kinetics or sub-saturated enzyme reactions. For highly saturated enzyme reactions, the flux is unaffected from the substrate pools but directly dependent on maximal velocity of the enzyme. As shown in Supplementary Table 1 and discussed later in detail (see section *Simplified model of the lipid II cycle* in Methods), the enzymes in the lipid II cycle operate on the brink of saturation, where the actual substrate pools still have a significant impact on the fluxes. Hence, the rate (k_i) at which a single molecule reacts from substrate state to product state is

$$k_{\mathrm{i}} = \frac{j_{\mathrm{PG}}}{[S_{\mathrm{i}}]},$$

and the dwell time (t_i) within the substrate state is given by

$$t_{\mathrm{i}} = \frac{1}{k_{\mathrm{i}}}.$$

Finally, the total time (t_{tot}) one carrier molecule needs to complete a full round of the cycle is the sum of all dwell times in the individual states

$$t_{\mathrm{tot}} = \sum t_{\mathrm{i}} = \sum \frac{1}{k_{\mathrm{i}}} = \sum \frac{[S_{\mathrm{i}}]}{j_{\mathrm{PG}}} = \frac{\sum [S_{\mathrm{i}}]}{j_{\mathrm{PG}}}.$$

Considering the PG synthesis rate of 1.01 x 10^5 molecules per minute and the sum of all lipid II carrier molecules in the individual states ($\sum[S_{\mathrm{i}}]$ ~1.5 x 10^5 molecules; Supplementary Table 1a), a full transport cycle requires ~90 seconds (Supplementary Fig. 1b).

In the full *in vivo* scenario, the *de novo* production of UPP in the cytoplasm and the dilution of all lipid II cycle intermediates create a non-trivial system of PG synthesis different from the considered closed-loop assumption (Supplementary Fig. 1a). In order to assess the validity of the principles derived from the closed-loop system for the *in vivo* scenario, we evaluated the impact of carrier (re-)cycling and *de novo* synthesis on PG synthesis. As detailed in the section below, the *de novo* synthesis of UPP balances the increasing demand of lipid carrier during bacterial growth. In particular, total amount of lipid II cycle intermediates has to be reproduced during one doubling time to satisfy the PG demand of both daughter cells. Accordingly, each individual lipid carrier molecule has to be produced once during one doubling time, that is within 36 min in *E. coli*. However, since a complete round takes approximately 1.5 minutes, each individual lipid carrier molecule can undergo 24 transport cycles within this time frame. Consequently, each lipid carrier is replenished by *de novo* synthesis due to cell growth not until 24 rounds of cycling, highlighting carrier recycling as the dominant process driving PG monomer transport across the cytoplasmic membrane. Thus, the assumption of a closed-loop system is a good approximation of the *in vivo* scenario, and can therefore help to rationalize the quantitative behavior of our full theoretical model of peptidoglycan biosynthesis.

Calibration of the mathematical model for *E. coli*

To identify physiologically relevant parameter values for the model in Eqs. (1-8) given in the Methods, we mined the literature for biochemical characterizations of the involved enzymes (Supplementary Table 1b). Since part of the reactions in the lipid II cycle are catalyzed by a single enzyme (e.g. MraY or MurG), the corresponding K_M values were directly used in the mathematical model. For all reactions catalyzed by several enzymes we found K_M values for at least one of the redundant enzymes, and, for parsimony reasons, fixed the parameters of our model to these values. In doing so, we assume that all PBPs and UppPs operate at similar, effective substrate levels determined by these K_M values. However, given that the enzyme catalytic rates, k_{cat}, as well as the precise enzyme abundances, [E], were only partially characterized (Supplementary Table 2), and that the effective flipping rates k_i were unknown altogether, we determined these missing parameters via a constrained optimization approach. To this end we optimized the maximal velocities ($v_{\max} = k_{\mathrm{cat}} \times [E]$) and k_i's for each reaction in Eqs. (1-8) given in the Methods, while matching 3 physiological constraints, as detailed in below, and later performing plausibility tests for the resulting parameter values. The physiological constraints are:

(i) While the precise distribution lipid II cycle intermediates in the outer and inner leaflet of the cytoplasmic membrane was unknown, the total pool levels (sum of outer and inner sub-pool) of all individual lipid carriers were determined experimentally during balanced growth (Supplementary Table 1a). This imposes constraints on the equilibrium values of the total lipid carrier pool levels in Model-Eqs. (1-8):

$$\mathrm{UPP_{IN}} + \mathrm{UPP_{OUT}} = \mathrm{UPP_{TOT}} = 1.2 \times 10^5 \text{ molecules per cell}$$
$$\mathrm{UP_{IN}} + \mathrm{UP_{OUT}} = \mathrm{UP_{TOT}} = 3.2 \times 10^4 \text{ molecules per cell}$$
$$\mathrm{LII_{IN}} + \mathrm{LII_{OUT}} = \mathrm{LII_{TOT}} = 1000 \text{ molecules per cell}$$
$$\mathrm{LI} = \mathrm{LI_{TOT}} = 700 \text{ molecules per cell}$$

(ii) During balanced growth, all fluxes j_i from one state of the cycle to the next have to be identical and need to equal the PG synthesis rate, j_{PG}. Consequently, flux balance requires that individual fluxes match the physiological estimate for the overall PG demand, i.e.

$$j_{\mathrm{i}} = v_{\max}\frac{[S_{\mathrm{i}}]}{K_{\mathrm{M}} + [S_{\mathrm{i}}]} = j_{\mathrm{PG}} = 1.01 \times 10^5 \text{ molecules per minute}$$

for the enzymatic reactions and

$$j_{\mathrm{i}} = k_{\mathrm{i}}[S_{\mathrm{i}}] = j_{\mathrm{PG}} = 1.01 \times 10^5 \text{ molecules per minute}$$

for the flipping reactions for UP and lipid II.

(iii) Given that UPP is synthesized in the cytoplasm and then fed into the cycle by flipping from the inner to the outer leaflet of the membrane, this flipping rate is not constrained by the rate of PG synthesis itself. However, if flipping of UPP to the external leaflet would be too slow, UPP carriers would be diluted by cell growth before entering the cycle. Therefore, it seemed plausible that cells flip UPP to the external leaflet at a rate comparable to *de novo* UPP synthesis in the cytoplasm. Under this constraint of 'efficient carrier usage', we demanded the rate of UPP flipping is equal to the rate of *de novo* synthesis required to keep the total lipid carrier pool $\sum[S_{\mathrm{i}}]$ at a constant level, i.e.

$$k_{\mathrm{UPP}}[\mathrm{UPP_{IN}}] = \alpha = \frac{\ln(2)}{T_{\mathrm{D}}}\sum[S_{\mathrm{i}}].$$

Ultimately, the parameters of the resulting set of equations and constraints were determined with a non-linear optimization approach (for further details of the algorithm see section *Parameter fitting algorithm*), providing an optimal set of parameters (Supplementary Table 2, Supplementary Fig. 1d), as well as an estimate for the variances and co-variances of individual parameters (Supplementary Fig. 1d). Furthermore, as the fitted parameters set the speed of the cycle reactions, they determine the distribution of the different lipid II cycle intermediates in the external and internal leaflet of the cytoplasmic membrane shown in the main text (Fig. 2a). The uncertainties in optimal parameters have only a moderate influence on the predicted distribution of individual lipid II cycle intermediate pool levels (Supplementary Fig. 4b), which in turn does not significantly affect our key results concerning the model predictions of the IC_{50} for different antibiotics (Supplementary Fig. 4a).

Quantitative considerations on peptidoglycan synthesis in *B. subtilis*

The PG demand of a single cell is determined by the size of the cell, in particular the cell surface area, and the number of PG layers that make up the cell wall. While the cell wall of *E. coli* comprises ~1.5 PG layers on average (1), *B. subtilis* features a PG thickness of about 20 layers (7), clearly leading to a raised demand of PG per unit of cell surface area in the latter. When further considering that at similar doubling times ($T_{\mathrm{D}}^{B.subtilis} = 40$ min vs. $T_{\mathrm{D}}^{E.coli} = 36$ min) the cell size and thereby cell surface area of *B. subtilis* is slightly higher compared to *E. coli* (Supplementary Table 3a), the theoretical demand of PG in *B. subtilis* is

$$\mathrm{PG}_{\mathrm{TOT}}^{B.subtilis} = \mathrm{PG}_{\mathrm{TOT}}^{E.coli} \times R_{\mathrm{T}} \times R_{\mathrm{A}},$$

with $R_{\mathrm{T}} = \frac{\text{cell wall thickness}^{B.subtilis}}{\text{cell wall thickness}^{E.coli}}$ defining the ratio between the thickness of the cell wall in Gram-positive and Gram-negative organisms and $R_{\mathrm{A}} = \frac{\text{surface area}^{B.subtilis}}{\text{surface area}^{E.coli}}$ taking the ratios of cell surface areas between both organisms into account. To further factor in the slight differences in generation times (Supplementary Table 3a), the PG synthesis rate in *B. subtilis* is

$$j_{\mathrm{PG}}^{B.subtilis} = \mathrm{PG}_{\mathrm{TOT}}^{B.subtilis} \times \frac{\ln(2)}{T_{\mathrm{D}}^{B.subtilis}}$$
$$= \mathrm{PG}_{\mathrm{TOT}}^{E.coli} \times R_{\mathrm{T}} \times R_{\mathrm{A}} \times \frac{\ln(2)}{T_{\mathrm{D}}^{B.subtilis}}$$
$$= j_{\mathrm{PG}}^{E.coli} \times R_{\mathrm{T}} \times R_{\mathrm{A}} \times \frac{1}{R_{\mathrm{G}}}$$

with $R_{\mathrm{G}} = \frac{T_{\mathrm{D}}^{B.subtilis}}{T_{\mathrm{D}}^{E.coli}}$. Ultimately, for *B. subtilis* this leads to a PG production rate of ~1.85 × 10^6 $\mathrm{molecules\ min}^{-1}$ (Supplementary Table 3a). To meet this higher PG demand we thus adapted all individual fluxes within the cycle, $j_{\mathrm{i}}^{B.subtilis}$, by the same factor, i.e.

$$j_{\mathrm{i}}^{B.subtilis} = j_{\mathrm{PG}}^{B.subtilis}.$$

As explained in the section *Kinetic model of the lipid II cycle* of the main text, the most parsimonious model consistent with all experimental observations assumes enzymes with higher catalytic rates and lower affinities to their substrates, as well as higher lipid II cycle intermediate pools to sufficiently saturate the enzymes. Hence, for the model in *B. subtilis* we increased the maximal velocities (v_{max}) of the individual enzymatic reactions proportional to the overall PG synthesis rate, to meet the increase of individual fluxes

$$E^{B.subtilis} \times k_{\mathrm{cat}}^{B.subtilis} = v_{\mathrm{max}}^{B.subtilis} = v_{\mathrm{max}}^{E.coli} \times R_{\mathrm{T}} \times R_{\mathrm{A}} \times \frac{1}{R_{\mathrm{G}}}.$$

Since at similar surface concentrations of enzymes, the slightly larger surface area of *B. subtilis* compared to *E. coli* (compare Fig. 2b, Supplementary Table 3b) suggests that the enzyme abundance scales according to

$$E^{B.subtilis} \sim E^{E.coli} \times R_{\mathrm{A}},$$

we demanded that the catalytic constants (k_{cat}) to increase in proportion to the remaining factors

$$k_{\mathrm{cat}}^{B.subtilis} = k_{\mathrm{cat}}^{E.coli} \times R_T \times \frac{1}{R_{\mathrm{G}}}.$$

Following the assumption of the speed/affinity tradeoff (see section *Kinetic model of the lipid II cycle*), the Michaelis constants K_{M} were raised by the same factor

$$K_{\mathrm{M}}^{B.subtilis} = K_{\mathrm{M}}^{E.coli} \times R_{\mathrm{T}} \times \frac{1}{R_{\mathrm{G}}}.$$

Finally, the need of higher lipid carrier concentrations in order to saturate the lower-affinity enzymes in *B. subtilis* demanded an increase in the UPP production rate α by a factor ($R_{\mathrm{T}} \times R_{\mathrm{A}} \times \frac{1}{R_{\mathrm{G}}}$). The resulting elevated lipid carrier concentrations proportionally raise the fluxes of the first order flipping reactions of UP, UPP and lipid II, such that the rate constants k_{i} had to remain unchanged.

Influence of antibiotic binding kinetics on the IC_{50}

While we extensively discussed the effect of the buffering factor $\left(1 + \widetilde{K}_{\mathrm{G}}\right)$ as the major contribution to the *in vivo* efficacy gap, there is a second factor arising from the difference between *in vivo* dissociation constant ($\widetilde{K}_{\mathrm{D}} = \frac{k_{\mathrm{diss}} + \gamma}{k_{\mathrm{ass}}}$) for the antibiotic-target interaction (Supplementary Table 4c). Compared to the *in vitro* dissociation constant ($K_{\mathrm{D}} = \frac{k_{\mathrm{diss}}}{k_{\mathrm{ass}}}$) this altered expression reflects a competition between the antibiotic binding reaction and the dilution of bound and unbound form target. If the antibiotic-bound

target is diluted before spontaneous dissociation (i.e. $k_{\text{diss}} << \gamma$), antibiotic action is less effective and the *in vivo* dissociation constant increases compared to the *in vitro* value. In contrast, if antibiotic-target interactions occur on much faster time scales than cell growth (i.e. k_{diss}, $k_{\text{ass}} >> \gamma$), the dilution of the pools of bound and unbound lipid intermediates has negligible impact on the efficacy of antibiotic action. This is indeed the case for the on-/off-kinetics (k_{diss} and k_{ass}) measured for the bacitracin/UPP interaction (Supplementary Table 4c, Supplementary Fig. 4c). Although there are no experimental data available for the binding kinetics of the other antibiotic-target interactions, simultaneously scaling k_{diss} and k_{ass} up and down by a factor β in our full model (while keeping their ratio $K_{\mathrm{D}} = \frac{k_{\mathrm{diss}}}{k_{\mathrm{ass}}}$ constant) reveals that the impact on the IC_{50} predictions is moderate (Supplementary Fig. 4d) and that the kinetic parameters chosen in our simulations (for $\beta = 1$) represent a conservative estimate for the *in vivo* shift of the IC_{50} (Supplementary Fig. 4d).

Supplementary Figures

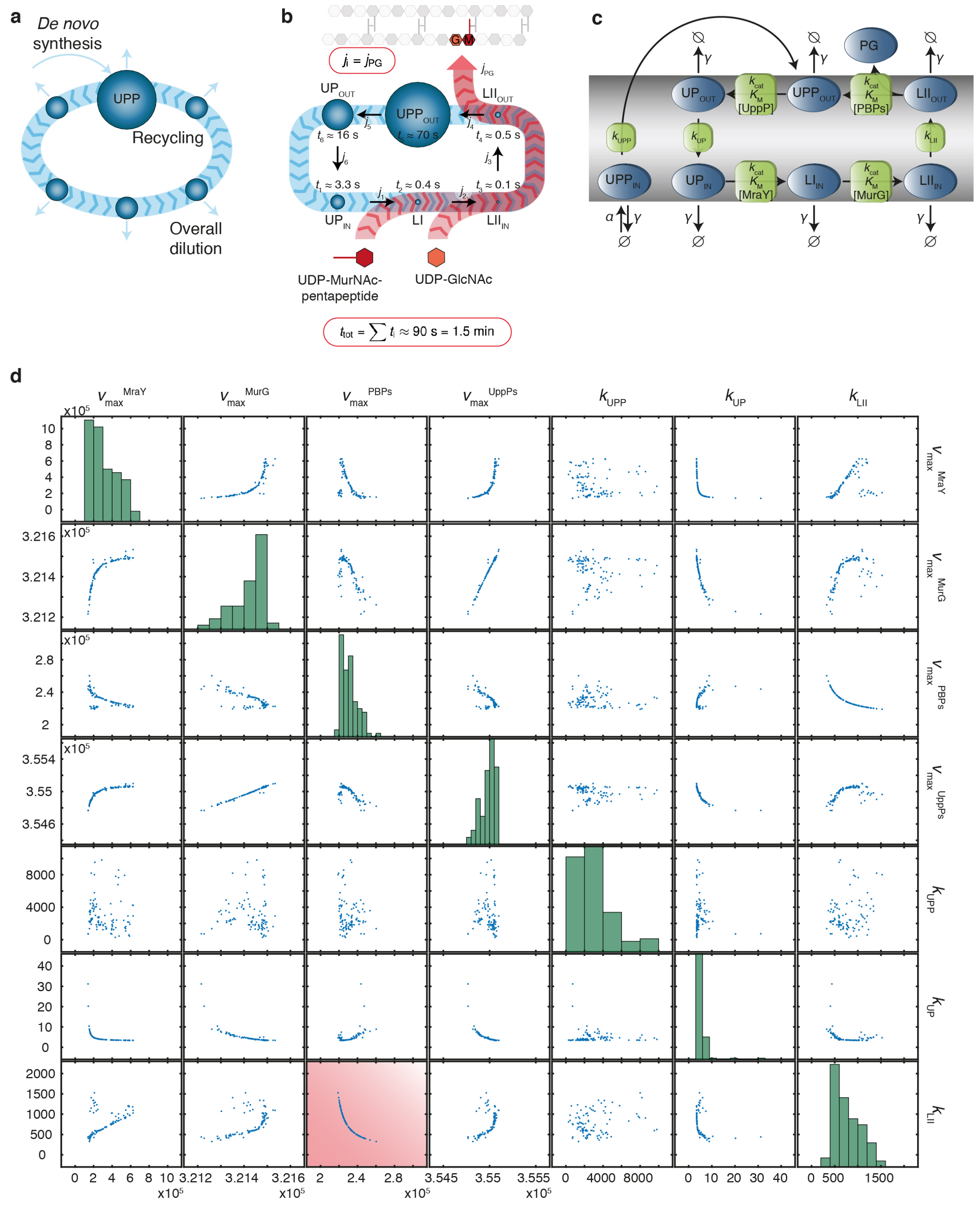

Supplementary Figure 1. Calibration of the theoretical model of the lipid II cycle in *E. coli* (a) The *de novo* synthesis of UPP balances the overall dilution of all lipid II cycle intermediates due to cell growth. However, as each individual lipid carrier molecule undergoes 24 rounds within the lipid II cycle before it is replenished by *de novo* synthesis, the (re-) cycling of lipid carrier is the dominant process that drives the synthesis of PG, as indicated by the thickness of the arrows. **(b)** Accordingly, the lipid II cycle can be considered as a closed-loop system with equilibrated fluxes $j_1,\ldots,j_6$ between the individual states of the system. In fact, all these fluxes equal the rate of PG synthesis, j_{PG}. Consequently, the dwell time of the individual molecules within one of the cycle states, which is dependent on the speed of the

reaction converting this molecule into the next state, can be calculated from the known overall rate of PG synthesis (see Supplementary Text for a detailed description). **(c)** The theoretical model quantifies the individual reactions of the lipid II cycle. The well-studied enzymatic reactions are parameterized by Michaelis-Menten kinetics, determined by the enzyme abundances (*[E]*), the catalytic rates of the enzymes (k_{cat}) as well as the substrate affinities to the enzymes (K_M). However, the less-studied flipping reactions are assumed to follow first order kinetics, defined by rate constants k_{UPP}, k_{UP} and k_{LII}, respectively. The production of UPP at rate α counterbalances the dilution of all lipid intermediates with rate γ, while $\frac{\alpha}{\gamma} = \sum[S_i]$ with S_i representing the intermediate substrate i in the cycle. **(d)** Since the rate constants of the flipping reactions, as well as the v_{max} values of the enzymatic reaction ($v_{max} = k_{cat} * [E]$), are largely unknown, we apply a constrained optimization approach based on the previous quantitative considerations to estimate the remaining parameters (see Supplementary Text for further details). The parameter sets of 100 independent parameter fits show variations in some of the parameter values. In fact, the most significant variations appear in the rate constants of the flipping reactions. In particular, a relation between the parameters of the flipping reactions and the upstream or downstream reactions exists – the product of both reaction rates is constant, that is a slower flipping reaction is predicted if the up- or downstream reaction is faster and *vice versa*. For instance, if the flipping of lipid II is fast, the lipid II pool accumulates at the outer leaflet of the membrane and the maximal velocity of the subsequent PBP-catalysed reaction is assumed to be slightly lower compared to a slower flipping because the higher substrate levels of lipid II also contribute partially to a higher flux (*red box*). This relation reflects the fact that the pool levels equilibrate in a way that all fluxes are identical. Consequently faster reactions immediately convert the substrate, leading to an accumulation of the product pool, which again displays the substrate pool for the subsequent reaction and thereby demands a reduced maximal velocity of this reaction for a balanced flux.

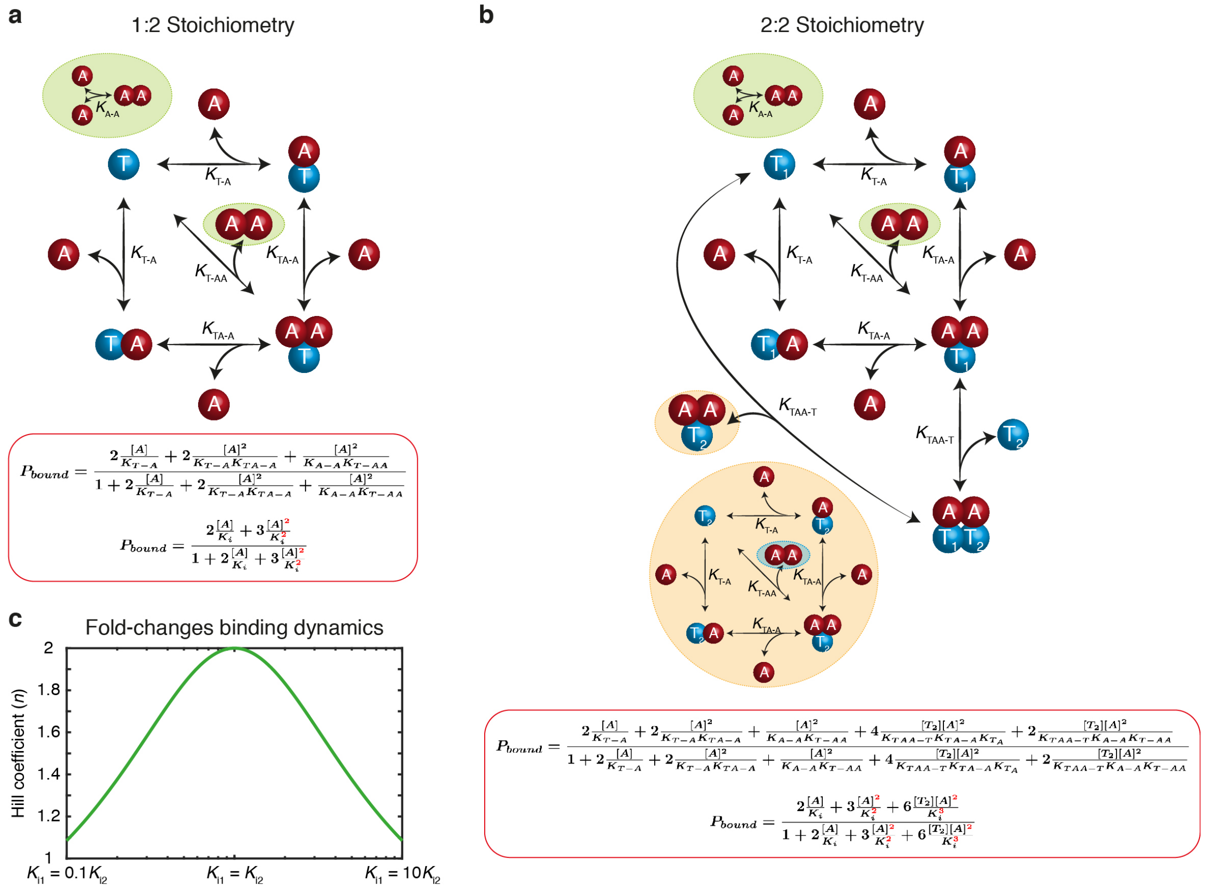

Supplementary Figure 2. Different multimeric binding scenarios lead to cooperative antibiotic-target-interaction. (a) In case of a two identical antibiotic molecules (A) interacting with one target molecule (T), the following binding events are conceivable: (i) The monomeric antibiotic binds to the target, determined by the dissociation constant $K_{T\text{-}A}$; (ii) the second antibiotic binds to the formed antibiotic-target-complex, determined by the dissociation constant $K_{TA\text{-}A}$ and (iii) the dimeric form of the antibiotic (dimer formation is determined by the dissociation constant $K_{A\text{-}A}$) directly interacts with target, determined by the dissociation constant $K_{T\text{-}AA}$. By evaluating all binding probabilities, we end up with a closed expression that describes the cumulative probability of a bound target P_{bound}, summarizing all possible forms of antibiotic-target-complexes (*red box, top*) (see Supplementary Text for calculation details). When assuming similar dissociation constants K_i for the individual interactions, the equation can be simplified and appears as the second one in the red box. Obviously, As Hill coefficients n>1 appear in the equation, the different binding scenarios can generate cooperativity in antibiotic-binding-interactions. **(b)** In the more complex scenario of two antibiotics interacting with two targets (T_1 and T_2), one further possible binding reaction arises, namely the binding of the second target to the complex formed by two antibiotic molecules and the other target molecule, which is determined by the dissociation constant $K_{TAA\text{-}T}$. Again, a closed expression describes the cumulative probability of a bound target P_{bound} (*red box, top*) and a simplified equation can be found, assuming similar K_i values for all binding reactions (*red box, bottom*). **(c)** Ultimately, evaluating the expression for the probability of a bound target, we also show that the Hill coefficient n takes the highest value if the dissociation constants of all binding reactions (K_{i1}, K_{i2}) are identical.

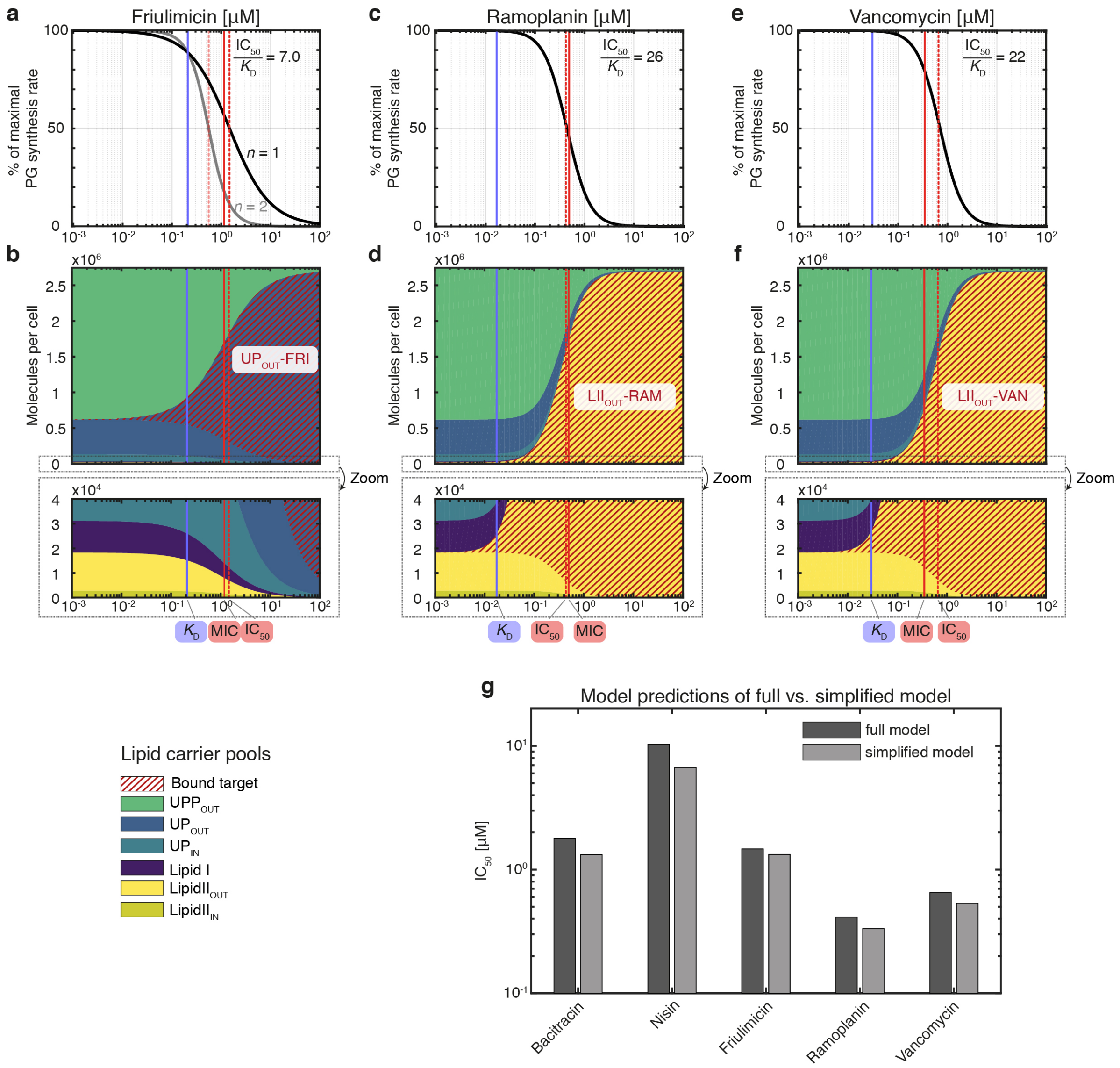

Supplementary Figure 3. Theory predicts a moderate *in vivo* efficacy gap for friulimicin and a more pronounced difference between IC_{50} and K_D for vancomycin and ramoplanin. (a, c, e) The mathematical model predicts the IC_{50} values (*red dashed lines*) for the non-cooperative (*grey*) and cooperative (*black*) binding of friulimicin to UP and the cooperative binding of ramoplanin and vancomycin to lipid II. The model predictions match the experimentally determined MICs (*red solid lines*) for all three antibiotics highly precise. In case of friulimicin, the comparison between the model predictions and the experimentally determined MIC supports the hypothesis of a non-cooperative binding to UP. **(b, d, f)** Lipid II cycle intermediate pools under different antibiotic concentrations as predicted by the theoretical model. While an efficient binding of the antibiotics to their target is always observable for antibiotic concentrations around the K_D values (*blue lines*), the reduction of the external levels of lipid II to 50% of its maximum - to achieve a halved PG synthesis rate - demands higher antibiotic concentrations (IC_{50}). However, as illustrated in the results section, this *in vivo* efficacy gap increases with smaller pool levels (LII<UP) and decreases with higher cooperativity (n = 2 for vancomycin and ramoplanin). **(g)** Taking similar pool levels distributions as well as comparable reactions rates as in the full model into account, the reduced model reproduces the predictions of the full model. The remaining differences arise from the different reaction kinetics in the two models, as elucidated in detail in the Supplementary Text.

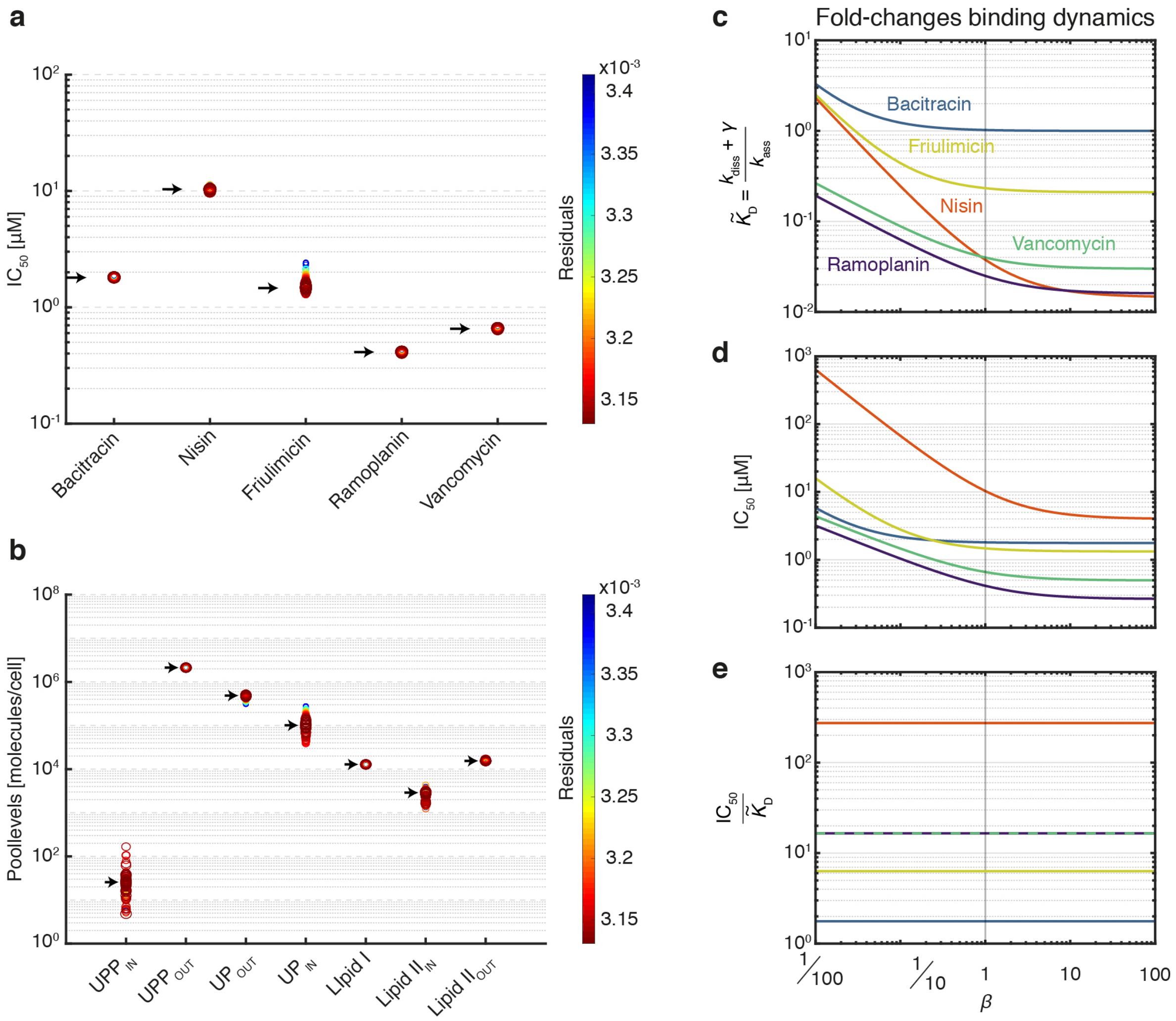

Supplementary Figure 4. Model predictions of the *in vivo* efficacy gap are robust against variations in the model parameters. (a, b) The repeatedly performed fitting procedure generates several close-to-optimal sets of model parameters, as characterized by the residuals χ^2. However, all parameter sets with $\chi^2 \leq 1.1 \times min\,(\chi^2)$ displayed here only lead to minor variations in the model predictions of the IC_{50} as well as the distribution of the individual pool levels. The most pronounced variation of the IC_{50} is observable for friulimicin, correlating with the shift between external and internal levels of UP. **(c, d, e)** Since the specific association- and dissociation rates of the different antibiotic-binding reactions (k_{diss} and k_{ass}, respectively) are unknown, we study the impact of faster and slower binding dynamics on the predictions of the IC_{50} by scaling k_{diss} and k_{ass} up and down by a factor β. The vertical grey lines identify the default model value $\beta = 1$. As the IC_{50} is a function of the *in vivo* dissociation constant ($\mathrm{IC}_{50} = \widetilde{K_\mathrm{D}}(1 + \widetilde{K_\mathrm{G}})$), variations in the *in vivo* dissociation constant ($\widetilde{K_\mathrm{D}}$) caused by an acceleration of the antibiotic binding reaction directly lead to variations in the IC_{50}. However, the *in vivo* dissociation constant ($\widetilde{K_\mathrm{D}}$) and thereby the model-predicted IC_{50} is robust against faster binding dynamics for most of the antibiotics, with nisin as an exception that features a slightly decreased *in vivo* dissociation constant ($\widetilde{K_\mathrm{D}}$) and consequently a reduced IC_{50} for an accelerated antibiotic binding and unbinding. Ultimately, the predicted buffering factor ($\frac{\mathrm{IC}_{50}}{\widetilde{K_\mathrm{D}}} = 1 + \widetilde{K_\mathrm{G}}$) is unaffected by fold-changes in the binding dynamics.

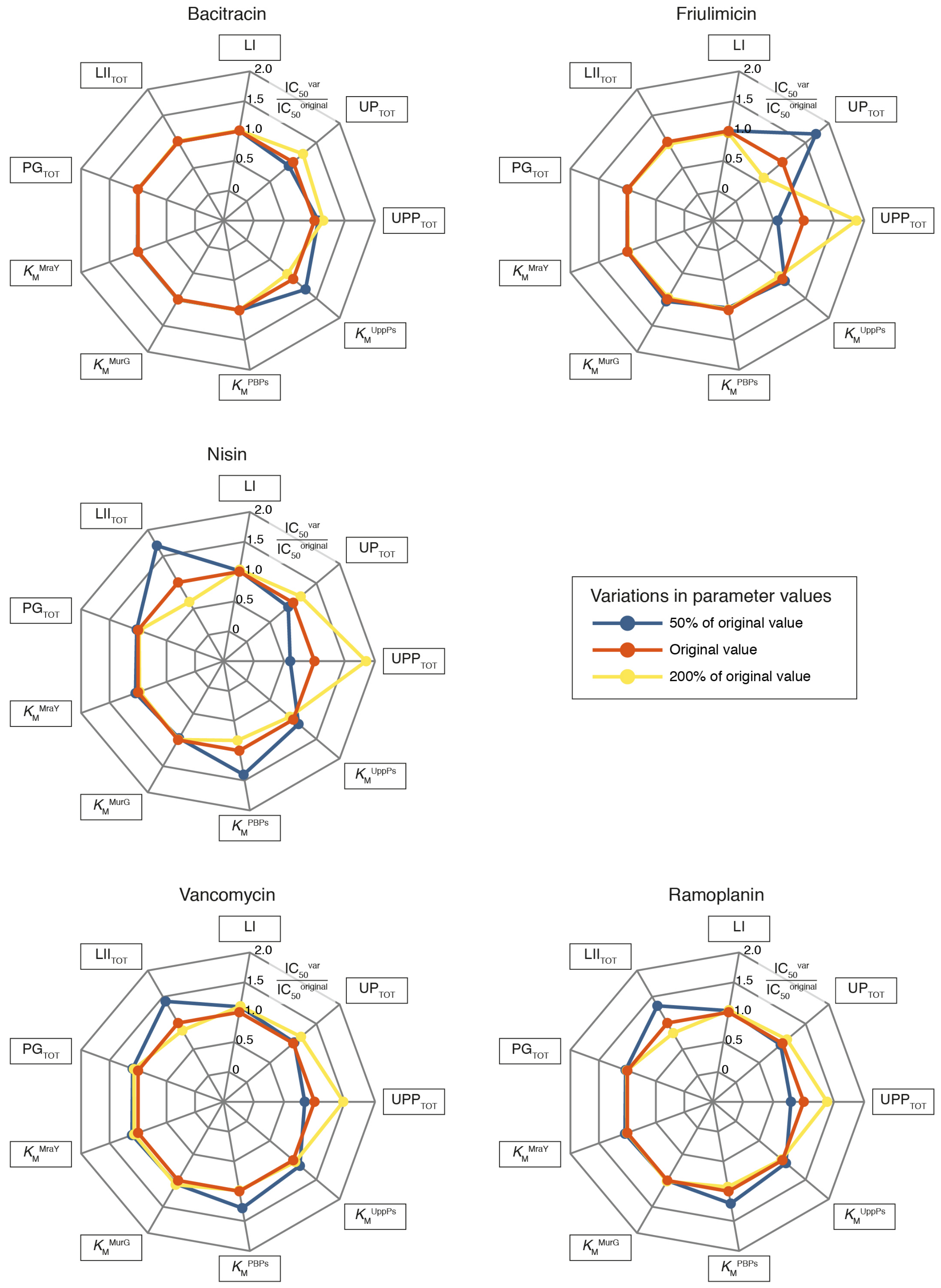

Supplementary Figure 5. Sensitivity of predicted IC_{50} values on changes of experimentally determined model parameters. The model includes experimentally measured K_M values as well as measurements of intermediate pools levels. Since the actual *in vivo* values of these parameters can differ from their *in vitro* measured quantities and can vary between different experimental setups, a sensitivity analysis of the model predictions against errors in the experimentally determined model parameters was performed. Here, the IC_{50} predictions for all 5 studied antibiotics were investigated upon

perturbation of each experimental parameter by a factor of two, i.e., for parameters taking 200% and 50% of the original parameter values given in Supplementary Table 1, respectively. For each parameter value, the relative deviation of the IC_{50} under parameter variation (IC_{50}^{var}) compared to the original model prediction ($IC_{50}^{original}$) is displayed on the respective axis of the spider's web. *Blue lines* indicate the deviations of the IC_{50} when the respective parameter value is reduced by a factor of two from the original value and *yellow lines* represent the results for two-fold increased parameter values compared to the original quantities. *Red lines* serve as references, as they illustrate the results based on the original parameter values. In general, the predictions of the IC_{50} for the 5 different antibiotics vary only sub-linearly under parameter variations. More precisely, two patterns are observable: (I) Changes in K_M values only affect the IC_{50} prediction for antibiotics that target the substrate of the corresponding reaction. For example, variations in K_M^{UppPs} lead to changes in bacitracin sensitivity and variations in K_M^{PBPs} cause variation in the IC_{50} of the lipid II-binding antibiotics (nisin, vancomycin and ramoplanin), respectively. These variations of the IC_{50} result from differences in the enzyme saturation according to the different K_M values, which has been shown to affect the IC_{50} as explained in the section *Reduced model of the lipid II cycle* in the Methods. (II) Variations in the pool size of UPP and the size of the target pool of each antibiotic affect the IC_{50} prediction of the respective antibiotic. In particular, a raised UPP pool (UPP_{TOT}) leads to higher IC_{50} predictions for friulimicin and the lipid II binding antibiotics and *vice versa*. Furthermore, an increase in the target pool levels of these antibiotics reduces the IC_{50} and lower target pools raise the IC_{50} respectively. These results once again highlight the influence of the buffering effect on the efficacy of the antibiotics that target the less-abundant intermediates in the cycle, such as friulimicin and especially the lipid II-binding antibiotics. While an increase of the UPP pool, which functions as the biggest reservoir in the lipid II cycle, supports the buffering effect and raises the IC_{50} consequently, an increase in the target pool size shifts the ratio between the reservoir and the target pool towards the target pool and reduces the buffering effect accordingly. Overall, all IC_{50}'s vary at maximum 2-fold under the analysed parameter variations and only deviations of orders of magnitudes from the experimentally determined quantities would lead to significant changes in the model predictions. Thus, the general conclusions of this study stay unaffected from moderate uncertainties in the experimentally determined model parameters.

Supplementary Tables

Supplementary Table 1: Model calibration

a) Pool levels of lipid II cycle intermediates in *E. coli*

Lipid intermediate	Pool level [molecules cell^{-1}]	Literature
**undecaprenyl phosphate (UP)**	3.21×10^4	(8)
**undecaprenyl pyrophosphate (UPP)**	1.16×10^5	(8)
**Lipid I**	700	(9)
**Lipid II**	1000	(9)
**UDP-MurNAc-pentapeptide**	1.75×10^5	(5)
**UDP-GlcNAc**	1.25×10^5	(6)
**total amount of PG in the sacculus**	3.5×10^6	(2)

b) Michaelis constants of lipid II cycle enzymes in *E. coli*

Enzyme	Substrate	K_M [µM]	Literature
**MraY**	UP	20	(10)
	UDP-MurNAc-pentapeptide	87	(11)
**MurG**	Lipid I	2.8	(12)
	UDP-GlcNAc	45	
**PBPs**	Lipid II	2	(13)
**UppPs**	UPP	530	(14)

Supplementary Table 2: Parameter fitting and validation

Reaction	Optimized parameter value	k_{cat} [min^{-1}] (literature)	Enzyme levels [molecules cell^{-1}] (calculated)	Enzyme levels [molecules cell^{-1}] [(d)] (literature)
**MraY**	v_{max} = 3.01 x 10^5 molecules min^{-1}			210-885
**MurG**	v_{max} = 3.21 x 10^5 molecules min^{-1}	560* [(a)]	573	168-518
**PBPs (PBP1A+ PBP1B+ RodA)**	v_{max} = 2.32 x 10^5 molecules min^{-1}	188 [(b)]	1234	358-1510 [(e)]
**UppPs**	v_{max} = 3.55 x 10^5 molecules min^{-1}	540 [(c)]	657	519-3215 [(f)]
**flipping UPP**	k_{UPP} = 1.84 x 10^3 min^{-1}			
**flipping UP**	k_{UP} = 3.81 min^{-1}			
**flipping lipid II**	k_{LII} = 642.23 min^{-1}			

(a) Ref: (15), *based on the importance of an intact membrane for the activity of MurG (16), we assumed a value 10-fold higher than measured *in vitro* for purified protein without membrane

(b) k_{cat} for the bifunctional transglycosylase PBP1b (17)

(c) k_{cat} measured for the undecaprenyl pyrophosphate phosphatase PgpB (14)

(d) Range of enzyme levels measured in (18) for *E. coli* doubling times of T_D = 56.3 min (lower enzyme levels) and T_D = 21.5 min (higher enzyme levels)

(e) Sum of PBP1A, PBP1B and RodA as the major penicillin-binding proteins with transglycosylase activity (19)

(f) Sum of all known undecaprenyl pyrophosphate phosphatases BacA, PgpB, YbjG and YeiU

Supplementary Table 3: Comparison between *E. coli* and *B. subtilis*

a) Cell size

Parameter	*E. coli*	Literature	*B. subtilis*	Literature
average cell length	3.27 µm	(20)	3.6 µm	(21)
average cell width	0.61 µm	(20)	0.86 µm	(21)
average cell area	6.26 μm^2 (a)		9.57 μm^2 (b)	
average cell volume	0.91 μm^3 (a)		1.89 μm^3 (b)	
PG thickness	1.5- fold layer	(1)	20-fold layer	(7)
PG	3.5×10^6 monomers $cell^{-1}$	(2)	7.13×10^7 monomers $cell^{-1}$ (d)	
generation time	36 min (LB)	(2)	40 min (CH medium)	(21)
PG turnover rate	0.5 (50%)	(3, 4)	0.5 (50%)	(22, 23)
PG synthesis rate	1.01×10^5 monomers $cell^{-1}$ (c)		1.85×10^6 monomers $cell^{-1}$ (c)	

(a) Calculated according to volume and area formula

$$V_{\text{rod}} = V_{\text{cyl}} + V_{\text{ball}} = \pi * \left(\frac{w}{2}\right)^2 * (l - w) + \frac{4}{3} * \pi * \left(\frac{w}{2}\right)^3$$

$$O_{\text{rod}} = M_{\text{cyl}} + O_{\text{ball}} = 2 * \pi * \left(\frac{w}{2}\right) * (l - w) + 4 * \pi * \left(\frac{w}{2}\right)^2$$

(b) Fit of cell size data from (21)

(c) $\frac{\text{PG monomers}}{\text{cell}} * (1 + \text{PG turnover rate}) * \frac{\ln(2)}{\text{generation time}}$

(d) Calculated according to scaling assumptions

$$\text{PG monomers per cell}^{B.subtilis} = \text{PG monomers per cell}^{E.coli} * \frac{thickness\ PG^{B.subtilis}}{thickness\ PG^{E.coli}} * \frac{surface\ area^{B.subtilis}}{surface\ area^{E.coli}}$$

b) Enzyme levels

Enzyme	Level in *E. coli* [molecules $cell^{-1}$] (20) (a)	Surface concentration [molecules μm^{-2}] (d)	Level in *B. subtilis* [molecules $cell^{-1}$] (26) (e)	Surface concentration [molecules μm^{-2}] (g)
MraY	210-885	44-74		
MurG	168-518	36-44	627	66
PBPs	358-1510 (b)	75-128	1227 (f)	128
UppPs	519-3215 (c)	109-270		
UppS/IspU	318-830	67-70	661	69

(a) Enzyme levels measured for two different growth rates (T_{D1} = 56.3 min and T_{D2} = 21.5 min)

(b) Sum of PBP1A, PBP1B and RodA as the major penicillin-binding proteins (19)

(c) Sum of all known undecaprenyl pyrophosphate phosphatases BacA, PgpB, YbjG and YeiU

(d) Considering a surface area of *E. coli* corresponding to the doubling times of enzyme measurements:

A = 4.37 μm^2 for a doubling time of T_{D1} = 56.3 min; A = 11.88 μm^2 for a doubling time of T_{D2} = 21.5 min (20)

(e) Enzyme levels measured in CH medium (T_D = 40 min)

(f) Sum of PBP1 and PBP 4 (only available data)

(g) Considering a surface area of *B. subtilis* of A = 9.57 μm^2 for a doubling time of T_D = 40 min (21)

c) Pool levels of lipid II cycle intermediates

**Intermediates**	**Surface concentration Gram-positives [molecules µm^{-2}]**	**Surface concentration Gram-negatives [molecules µm^{-2}]**	**Ratio**	**Literature**
**UP**	9.2 ($\pm$ 3.1) x 10^4 *(M. flavus)*<br>11.5 ($\pm$ 3.8) x 10^4 *(L. monocytogenes)*	5.1 x 10^3 *(E. coli)*	18-22	(8, 25)
**UPP + UP**	3.3 x 10^5 *(S. aureus)*	2.4 x 10^4 *(E. coli)*	14	(8)
**Lipid II**	2.0 x 10^3 *(B. megaterium)*	160 *(E. coli)*	12	(9, 26)

Supplementary Table 4: Antibiotic activity in *B. subtilis*

a) K_D values and cooperativity of antibiotic-target-interaction

Antibiotic	Target	K_D [μM]	Cooperativity (n)	Literature
**bacitracin**	UPP	1	1	(27)
**friulimicin**	UP	0.21		(28)
**ramoplanin**	Lipid II	0.016	2	(29)
**vancomycin**	Lipid II	0.03	2	(30, 31)
**nisin**	Lipid II	0.015	1	(32, 33)

b) MIC values of antibiotic-target-interaction

Antibiotic	Strain	MIC [μM]	Method	Medium	Literature
**bacitracin**	*B. subtilis* (W168*ΔbceAB*)	1.67	E-test	Müller-Hinton (MH)	(34)
	S. aureus (NCTC 8325 rsbU$^+$ *ΔvraDEΔbceAB)*	4.2	killing curve assay	Trypticase Soy Broth (TSB)	(35)
	E. faecalis (JH2-2 *ΔEF2050-EF2049 ΔEF2752-EF2751)*	5.6	broth dilution assay	MH	(36)
**friulimicin**	*B. subtilis* (W168)	1.15	killing curve assay	Luria-Bertani (LB)	(37)
**ramoplanin**	*B. subtilis* (PY79)	0.49	broth dilution assay	LB	(38)
**vancomycin**	*B. subtilis* (ATCC 6633)	0.35	broth dilution assay	TSB	(39)
	S. aureus (RN4220 ΔvraFG)	0.7	broth dilution assay	TSB	(40)
	E. faecalis (VSE)	1.4	broth dilution assay and E-test	MH	(41)
**nisin**	*B. subtilis* (W168*ΔpsdAB*)	4.77	killing curve assay	MH	(42)
	S. aureus (NCTC 8325 rsbU$^+$ *ΔvraDEΔbceAB)*	1.2	killing curve assay	TSB	(35)

c) *In vivo* K_D values of antibiotic-target-interaction

Antibiotic	Target	$\widetilde{K_D}$ [μM]
**bacitracin**	UPP	1.02
**friulimicin**	UP	0.23
**ramoplanin**	Lipid II	0.025
**vancomycin**	Lipid II	0.04
**nisin**	Lipid II	0.038

Supplementary References

(1) Labischinski, H., Goodell, E.W., Goodell, A. & Hochberg, M.L. Direct proof of a more-than-single-layered peptidoglycan architecture of *Escherichia coli* W7: a neutron small-angle scattering study. *Journal of Bacteriology* **173**, 751–756 (1991).

(2) Mengin-Lecreulx, D. & van Heijenoort, J. Effect of growth conditions on peptidoglycan content and cytoplasmic steps of its biosynthesis in *Escherichia coli*. *Journal of Bacteriology* **163**, 208–212 (1985).

(3) Goodell, E.W. & Schwarz, U. Release of cell wall peptides into culture medium by exponentially growing *Escherichia coli*. *Journal of Bacteriology* **162**, 391–397 (1985).

(4) Goodell, E.W. Recycling of murein by *Escherichia coli*. *Journal of Bacteriology* **163**, 305–310 (1985).

(5) Mengin-Lecreulx, D., Flouret, B. & van Heijenoort, J. Cytoplasmic steps of peptidoglycan synthesis in *Escherichia coli*. *Journal of Bacteriology* **151**, 1109–1117 (1982).

(6) Mengin-Lecreulx, D., Flouret, B. & van Heijenoort, J. Pool levels of UDP N-acetylglucosamine and UDP N-acetylglucosamine-enolpyruvate in *Escherichia coli* and correlation with peptidoglycan synthesis. *Journal of Bacteriology* **154**, 1284–1290 (1983).

(7) Breukink, E. & de Kruijff, B. Lipid II as a target for antibiotics. *Nat Rev Drug Discov* **5**, 321–332 (2006).

(8) Barreteau, H. *et al*. Quantitative high-performance liquid chromatography analysis of the pool levels of undecaprenyl phosphate and its derivatives in bacterial membranes. *J Chromatogr B Analyt Technol Biomed Life Sci* **877**, 213–220 (2009).

(9) van Heijenoort, Y., Gómez, M., Derrien, M., Ayala, J. & van Heijenoort, J. Membrane intermediates in the peptidoglycan metabolism of *Escherichia coli*: possible roles of PBP 1b and PBP 3. *Journal of Bacteriology* **174**, 3549–3557 (1992).

(10) Brandish, P.E. *et al*. Slow binding inhibition of phospho-N-acetylmuramyl-pentapeptide-translocase (*Escherichia coli*) by mureidomycin A. *Journal of Biological Chemistry* **271**, 7609–7614 (1996).

(11) Geis, A. & Plapp, R. Phospho-N-acetylmuramoyl-pentapeptide-transferase of *Escherichia coli* K12. Properties of the membrane-bound and the extracted and partially purified enzyme. *Biochimica et Biophysica Acta (BBA) - Biomembranes* **527**, 414–424 (1978).

(12) Crouvoisier, M., Auger, G., Blanot, D. & Mengin-Lecreulx, D. Role of the amino acid invariants in the active site of MurG as evaluated by site-directed mutagenesis. *Biochimie* **89**, 1498–1508 (2007).

(13) Schwartz, B., Markwalder, J.A., Seitz, S.P., Wang, Y. & Stein, R.L. A kinetic characterization of the glycosyltransferase activity of *Eschericia coli* PBP1b and development of a continuous fluorescence assay. *Biochemistry* **41**, 12552–12561 (2002).

(14) Touzé, T., Blanot, D. & Mengin-Lecreulx, D. Substrate specificity and membrane topology of *Escherichia coli* PgpB, an undecaprenyl pyrophosphate phosphatase. *Journal of Biological Chemistry* **283**, 16573–16583 (2008).

(15) Auger, G., van Heijenoort, J., Mengin-Lecreulx, D. & Blanot, D. A MurG assay which utilises a synthetic analogue of lipid I. *FEMS Microbiology Letters* **219**, 115–119 (2003).

(16) Müller, A. *et al.* Daptomycin inhibits cell envelope synthesis by interfering with fluid membrane microdomains. *P Natl Acad Sci Usa* **113**, E7077–E7086 (2016).

(17) Sung, M.-T. *et al.* Crystal structure of the membrane-bound bifunctional transglycosylase PBP1b from *Escherichia coli*. *P Natl Acad Sci Usa* **106**, 8824–8829 (2009).

(18) Li, G.-W., Burkhardt, D., Gross, C.A. & Weissman, J.S. Quantifying absolute protein synthesis rates reveals principles underlying allocation of cellular resources. *Cell* **157**, 624–635 (2014).

(19) Typas, A., Banzhaf, M., Gross, C.A. & Vollmer, W. From the regulation of peptidoglycan synthesis to bacterial growth and morphology. *Nat Rev Micro* **10**, 123–136 (2012).

(20) Taheri-Araghi, S. *et al.* Cell-size control and homeostasis in bacteria. *Curr Biol* **25**, 385–391 (2015).

(21) Sharpe, M.E., Hauser, P.M., Sharpe, R.G. & Errington, J. *Bacillus subtilis* cell cycle as studied by fluorescence microscopy: constancy of cell length at initiation of DNA replication and evidence for active nucleoid partitioning. *Journal of Bacteriology* **180**, 547–555 (1998).

(22) Mauck, J. & Glaser, L. Turnover of the cell wall of *Bacillus subtilis* W-23 during logarithmic growth. *Biochemical and Biophysical Research Communications* **39**, 699–706 (1970).

(23) Mauck, J., Chan, L. & Glaser, L. Turnover of the cell wall of Gram-positive bacteria. *Journal of Biological Chemistry* **246**, 1820–1827 (1971).

(24) Muntel, J. *et al.* Comprehensive absolute quantification of the cytosolic proteome of *Bacillus subtilis* by data independent, parallel fragmentation in liquid chromatography/mass spectrometry (LC/MS(E)). *Mol Cell Proteomics* **13**, 1008–1019 (2014).

(25) Kramer, N.E. *et al*. Resistance of Gram-positive bacteria to nisin is not determined by lipid II levels. *FEMS Microbiology Letters* **239**, 157–161 (2004).

(26) Fuchs-Cleveland, E. & Gilvarg, C. Oligomeric intermediate in peptidoglycan biosynthesis in *Bacillus megaterium*. *Proc Natl Acad Sci USA* **73**, 4200–4204 (1976).

(27) Storm, D.R. & Strominger, J.L. Complex formation between bacitracin peptides and isoprenyl pyrophosphates. The specificity of lipid-peptide interactions. *Journal of Biological Chemistry* **248**, 3940–3945 (1973).

(28) Reder-Christ, K. *et al*. Model membrane approaches to determine the role of calcium for the antimicrobial activity of friulimicin. *Int J Antimicrob Agents* **37**, 256–260 (2011).

(29) Hu, Y., Helm, J.S., Chen, L., Ye, X.-Y. & Walker, S. Ramoplanin inhibits bacterial transglycosylases by binding as a dimer to lipid II. *J Am Chem Soc* **125**, 8736–8737 (2003).

(30) Beauregard, D.A., Maguire, A.J., Williams, D.H. & Reynolds, P.E. Semiquantitation of cooperativity in binding of vancomycin-group antibiotics to vancomycin-susceptible and -resistant organisms. *Antimicrobial Agents and Chemotherapy* **41**, 2418–2423 (1997).

(31) Schäfer, M., Schneider, T. & Sheldrick, G.M. Crystal structure of vancomycin. *Structure* **4**, 1509–1515 (1996).

(32) Wiedemann, I. *et al*. Specific binding of nisin to the peptidoglycan precursor lipid II combines pore formation and inhibition of cell wall biosynthesis for potent antibiotic activity. *Journal of Biological Chemistry* **276**, 1772–1779 (2001).

(33) 't Hart, P., Oppedijk, S.F., Breukink, E. & Martin, N.I. New insights into nisin's antibacterial mechanism revealed by binding studies with synthetic lipid II analogues. *Biochemistry* **55**, 232–237 (2016).

(34) Radeck, J. *et al*. Anatomy of the bacitracin resistance network in *Bacillus subtilis*. *Molecular Microbiology* **100**, 607–620 (2016).

(35) Hiron, A., Falord, M., Valle, J., Débarbouillé, M. & Msadek, T. Bacitracin and nisin resistance in *Staphylococcus aureus*: a novel pathway involving the BraS/BraR two-component system

(SA2417/SA2418) and both the BraD/BraE and VraD/VraE ABC transporters. *Molecular Microbiology* **81**, 602–622 (2011).

(36) Gebhard, S. *et al.* Identification and characterization of a bacitracin resistance network in *Enterococcus faecalis*. *Antimicrobial Agents and Chemotherapy* **58**, 1425–1433 (2014).

(37) Wecke, T. *et al.* Daptomycin versus Friulimicin B: in-depth profiling of *Bacillus subtilis* cell envelope stress responses. *Antimicrobial Agents and Chemotherapy* **53**, 1619–1623 (2009).

(38) Tiyanont, K. *et al*. Imaging peptidoglycan biosynthesis in *Bacillus subtilis* with fluorescent antibiotics. *Proc Natl Acad Sci USA* **103**, 11033–11038 (2006).

(39) Mota-Meira, M., LaPointe, G., Lacroix, C. & Lavoie, M.C. MICs of mutacin B-Ny266, nisin A, vancomycin, and oxacillin against bacterial pathogens. *Antimicrobial Agents and Chemotherapy* **44**, 24–29 (2000).

(40) Yoshida, Y. *et al*. Bacitracin sensing and resistance in *Staphylococcus aureus*. *FEMS Microbiology Letters* **320**, 33–39 (2011).

(41) Shaaly, A., Kalamorz, F., Gebhard, S. & Cook, G.M. Undecaprenyl pyrophosphate phosphatase confers low-level resistance to bacitracin in *Enterococcus faecalis*. *J Antimicrob Chemother* **68**, 1583–1593 (2013).

(42) Staroń, A., Finkeisen, D.E. & Mascher, T. Peptide antibiotic sensing and detoxification modules of *Bacillus subtilis*. *Antimicrobial Agents and Chemotherapy* **55**, 515–525 (2011).
